# Association of *CDH11* with Autism Spectrum Disorder Revealed by Matched-gene Co-expression Analysis and Mouse Behavioral Studies

**DOI:** 10.1007/s12264-021-00770-0

**Published:** 2021-09-14

**Authors:** Nan Wu, Yue Wang, Jing-Yan Jia, Yi-Hsuan Pan, Xiao-Bing Yuan

**Affiliations:** 1 Key Laboratory of Brain Functional Genomics of Shanghai and the Ministry of Education, Institute of Brain Functional Genomics, School of Life Science and the Collaborative Innovation Center for Brain Science, East China Normal University, Shanghai, 200062 China; 2 Hussman Institute for Autism, Baltimore, 21201 USA; 3 Department of Anatomy and Neurobiology, University of Maryland School of Medicine, Baltimore, 21201 USA

**Keywords:** *CDH11*, Autism spectrum disorder, Gene co-expression analysis, Matched-gene co-expression analysis

## Abstract

A large number of putative risk genes for autism spectrum disorder (ASD) have been reported. The functions of most of these susceptibility genes in developing brains remain unknown, and causal relationships between their variation and autism traits have not been established. The aim of this study was to predict putative risk genes at the whole-genome level based on the analysis of gene co-expression with a group of high-confidence ASD risk genes (hcASDs). The results showed that three gene features – gene size, mRNA abundance, and guanine-cytosine content – affect the genome-wide co-expression profiles of hcASDs. To circumvent the interference of these features in gene co-expression analysis, we developed a method to determine whether a gene is significantly co-expressed with hcASDs by statistically comparing the co-expression profile of this gene with hcASDs to that of this gene with permuted gene sets of feature-matched genes. This method is referred to as "matched-gene co-expression analysis" (MGCA). With MGCA, we demonstrated the convergence in developmental expression profiles of hcASDs and improved the efficacy of risk gene prediction. The results of analysis of two recently-reported ASD candidate genes, *CDH11* and *CDH9,* suggested the involvement of *CDH11*, but not *CDH9*, in ASD. Consistent with this prediction, behavioral studies showed that *Cdh11*-null mice, but not *Cdh9*-null mice, have multiple autism-like behavioral alterations. This study highlights the power of MGCA in revealing ASD-associated genes and the potential role of *CDH11* in ASD.

**Supplementary Information:**

The online version contains supplementary material available at 10.1007/s12264-021-00770-0.

## Introduction

Autism spectrum disorder (ASD) is a heterogeneous neurodevelopmental condition with a complex genetic basis [1, 2]. A large number of putative risk genes have been identified by genetic linkage analyses, genome-wide association studies, whole-exome sequencing, or whole-genome sequencing [3–5]. However, the functions of most of these putative risk genes in the developing brain remain unknown. For some novel risk genes, the genetic evidence supporting their association with ASD is not sufficient. Therefore, causal relationships between the variations of many risk genes and autism traits have not been established. In order to prioritize the investigation of genes and signaling pathways of high relevance to ASD, a method to determine the functional importance of a large group of putative risk genes is vital.

**Fig. 1:**
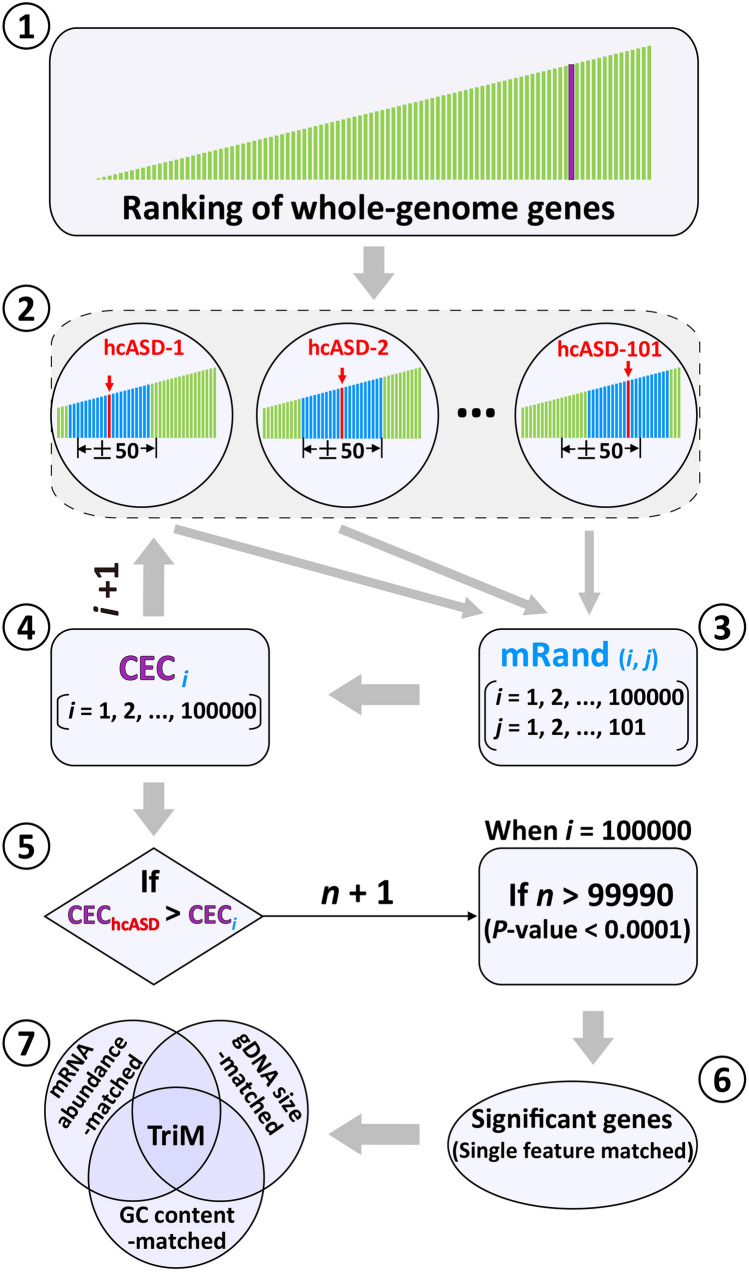
Flowchart of matched gene co-expression analysis (MGCA). Whole-genome genes are ranked by their features, creating various gene lists ①. For each gene in the hcASD set (hcASD-1, hcASD-2, ⋅⋅⋅ to hcASD-101), a feature-matched gene is randomly selected from the range of 50 genes above and 50 below (±50 range) this gene in the ranked lists ② to generate a matched random gene set (mRand) ③. For each gene in the whole genome, its co-expression coefficient (CEC) with each of the mRand gene set (CEC_i_) is computed ④, and 100,000 permutations are conducted (*i* = 1, 2, … 100,000). Each CEC_i_ is compared to the CEC of the same gene with the hcASD gene set (CEC_hcASD_). The number of permutations with CEC_i_ < CEC_hcASD_ (*n* starts from 0 for each gene evaluated, *n* + 1 if CEC_i_ < CEC_hcASD_) is counted ⑤. After 100,000 permutations, genes with *n* >99,990, which correspond to a permutation *P* < 0.0001, are considered significantly co-expressed with hcASDs under a feature-matched condition ⑥. Genes significantly co-expressed with hcASDs under all three feature-matched conditions are defined as Triple-matched genes (TriM) ⑦.

The highly diverse ASD risk genes are believed to functionally converge on several common molecular pathways closely related to ASD, such as the Wnt signaling pathway, the mammalian target of rapamycin pathway, and dendritic development and synaptic remodeling pathways [3, 6]. Consistent with the functional convergence of ASD risk genes, several studies have shown the convergence of the developmental expression profiles of a large group of risk genes [7, 8]. It is generally believed that genes with similar expression profiles are co-regulated or have related functions [7, 9]. The co-expression of genes within a biological pathway is a strong indication of their shared functions [9]. Based on this concept, computational analyses of various brain transcriptomes have been conducted to identify potential co-expression networks of ASD risk genes and to discover brain circuits that may be affected by risk genes [7, 8, 10, 11]. In these studies, the correlation coefficient (CC) of a pair of genes is calculated based on their expression levels in different brain regions or at different developmental stages. Genome-wide gene co-expression networks have been constructed by setting an empirically determined CC threshold [7]. Genetic mutation and protein interaction data have also been incorporated into gene co-expression analysis (GCA) along with novel data analysis algorithms, such as machine learning, to predict putative ASD risk genes and the molecular pathways on which they converge [12]. A major limitation in most of these studies is the lack of ways to overcome the potential effects of confounding factors, such as the size, expression level (mRNA abundance), and guanine-cytosine (GC) content of genes, on the results of GCA [13]. Most ASD risk genes are large and have a higher expression level in the brain than in other tissues [14]. It is unclear whether the size or expression level of an ASD gene affects its co-expression with other genes. It is also unclear whether the convergent pattern of developmental expression profiles is specific to ASD risk genes or a common property of genes with similar features, such as large size and high mRNA abundance [13]. Therefore, we developed a new method called “matched-gene co-expression analysis” (MGCA) (Fig. 1) to examine whether a gene exhibits significant co-expression with a group of high-confidence ASD risk genes (hcASDs) independent of confounding gene features.

## Materials and Methods

### Data Filtering and Computation of Co-expression Coefficients

The human brain transcriptome dataset from BrainSpan (www.brainspan.org) (RNA-Seq Gencode v10) was used for GCA. This dataset contained 256 transcriptomes of 16 different brain regions. The developmental stages ranged from post-conception week 8 (PCW8) to 40 years old (40Y). Normalized mRNA expression values were represented by RPKM (reads per kilobase per million mapped reads). The average mRNA expression level of each gene in all tissues was considered to be the mRNA abundance level of a gene. Gene lengths were determined based on annotations provided by the National Center for Biotechnology Information. The GC content of a gene was obtained from Ensembl Genome Browser. Based on statistical analyses of genetic data described previously, 101 risk genes that reached a genome-wide significance threshold (false discovery rate, FDR ≤0.1) [15] were used as the hcASD gene set (high confidence ASD risk gene set; Table S1). Genes with an abundance level lower than the lowest abundance level of hcASDs were filtered out (Table S1). Perl scripts were written to run most calculations. Pair-wise Pearson’s CC was used to indicate the tendency for co-expression of a gene pair. Heatmaps were constructed with the software R based on the CC matrix of 1/100 evenly-distributed genes. The mean CC was defined as the co-expression coefficient (CEC), which indicates the tendency for co-expression of a gene with a specific set of genes ($CEC = \frac{1}{M} \sum_{i = 1}^{M}{CC}_{ i }, i = 1, 2, \dots ,M$; where *M* is the total gene number of a gene set) or the tendency of co-expression of two gene sets [$CEC = \frac{1}{N*M} \sum_{k = 1}^{N}{(\sum_{i = 1}^{M}{CC}_{k i})}, k = 1, 2, \dots ,N; i = 1, 2, \dots ,M$; where *M* and *N* represent the total gene number of two different gene sets].

### Gene Set Definition

After data filtering, a total of 12,250 genes with available information on gene length, mRNA level, and GC content were identified and used for the study (Table S1). In addition to the hcASD gene set, the following gene sets were used: cASD (combined ASD genes), mRand (matched random genes), Rand (random genes), TriM (triple-matched genes), Top (top-ranked genes by CEC value), TriM-only (genes only in the TriM gene set), and Top-only (genes only in the Top gene set). The cASD gene set was a combined set of ASD-associated genes containing 514 non-redundant genes from nine different sets of previously-reported ASD-associated genes (Table S5) [7, 15–21]. Each mRand gene set contained 101 genes, and one of the three features of each gene was matched with that of the corresponding hcASD gene in the hcASD gene set. To generate the mRand gene sets, each feature-matched gene was randomly selected within the ±50 range of the corresponding hcASD gene in the ranked gene list using a Perl Script. Each Rand gene set contained 101 genes randomly selected from the whole gene list using a Perl Script without considering matched gene features. TriM was the set of genes revealed by matched-gene co-expression analysis (MGCA) that exhibited significant co-expression with the hcASD gene set under all three matched conditions (Figs 1, 4A). The Top gene set contained top-ranked genes with the highest CEC values with hcASD. The TriM-only and Top-only gene sets contained non-overlapped genes present only in the TriM or the Top gene set.

### Gene Ontology (GO) Analysis

GO analysis was performed using DAVID v6.8 (http://david.ncifcrf.gov/tools.jsp), and the human whole-genome genes provided by DAVID were used as the background list. A corrected *P-*value of 0.05 (Benjamini–Hochberg method) was used.

### Pathway Enrichment Analysis

Metascape (http://metascape.org) was used for pathway enrichment analysis and to draw heatmaps. Pairwise similarities between any two significant terms were computed based on Kappa-test scores. The enriched terms were then hierarchically clustered into a tree with a kappa score of 0.3 as the threshold.

### Ethics Approval

Animal care and handling were performed according to the guidelines for the Care and Use of Laboratory Animals of the National Institutes of Health. All animal experiments were approved by the Animal Care and Use Committees of Hussman Institute for Autism (06012015D), University of Maryland School of Medicine (0515017), and East China Normal University (m20190236).

### Animals

*Cdh9*-null mice (*Cdh9*^Laz^, C57BL/6-ICR mixed background) [22] were provided by Dr. Joshua R Sanes at Harvard University. *Cdh11*-null mice [23] were from the Jackson Labs (*Cdh11*^*tm1Mta*^/HensJ, https://www.jax.org/strain/023494, C57BL/6-129Sv-CD-1 mixed background). All mice were housed in groups of five with free access to food and water and kept on a 12-h light/dark cycle. Mice were tattooed on the tail using fluorescent ink for identification. A UV flashlight was used to visualize the tattooed identification numbers. All behavioral tests were conducted during the daytime on mice 2–5 months of age. The experimenter was blind to the genotype of the animal during behavioral experiments. The surface of the apparatus for behavioral tests was cleaned with 50% ethanol between tests. At least 5 min between cleaning and the next test was allowed for ethanol evaporation and odor dissipation.

### Genotyping

Genotyping of *Cdh9*-null mice was done by PCR as previously described [22]. The PCR product for the wild-type (WT) *Cdh9* allele was 550 bp amplified with the primer pair *Cdh9*-P1 (CCA CTA CAG GAA ACC TTT GGG TT) and *Cdh9*-P3 (ATG CAA ACC ATC AGG TAT ACC AAC C), and that of the mutant allele was 430 bp amplified with the primer pair *Cdh9*-P1 and *Cdh9*-P2 (CGT GGT ATC GTT ATG CGC CT). The annealing temperature for *Cdh9* PCRs was 63°C. For genotyping of *Cdh11*-null mice, the primer pair *Cdh11*-P1 (CGC CTT CTT GAC GAG TTC) and *Cdh11*-P2 (CAC CAT AAT TTG CCA GCT CA) were used for amplification of the mutant allele, and the primer pair *Cdh11*-P3 (GTT CAG TCG GCA GAA GCA G) and *Cdh11*-P2 were used for the WT allele. The annealing temperatures for PCR were 63.1°C and 56°C for the mutant and WT alleles, respectively. The sizes of the PCR products for the mutant and WT alleles were 500 bp and 400 bp, respectively.

### Behavioral Tests

Mice 3–5 months old were used for the behavioral tests. Animals were handled before the test (10 min/day for 3 days). The general order of tests was the open field test, elevated plus maze, sociability test, rotarod test, and gripping force test. Animals rested for at least 3 days after finishing one test. During all tests, the experimenter was blind to mouse genotype. Three batches of mice were analyzed, and data were pooled for analysis.

#### Open Field Test

The test mouse was allowed to freely explore the open field arena (50 cm × 50 cm) for 30 min. The movement was videoed and tracked by an automated tracking system (EthoVision XT 11.5, Noldus Information Technology, Leesburg, USA), which also recorded rearing, hopping, turning, self-grooming, movement time, total distance moved, and time spent in the center of the arena (1/2 of total size).

#### Elevated Plus Maze Test (EPM)

The standard EPM apparatus consisted of two open and two closed arms, 30 cm × 5 cm each, connected by a central platform (5 cm × 5 cm). The maze was 30 cm above the floor. The test mouse was gently placed on the central platform with its head facing one closed arm and was allowed to freely explore for 10 min. The time spent in the open arms and the number of open arm entries were recorded.

#### Grip Strength Test

The test mouse was placed on a metal grid on top of a transparent chamber. The grid was quickly inverted, and the time for the mouse to drop off the grid was noted. Five consecutive trials were carried out, and the average hanging time for each mouse was calculated. The maximum hanging time was set to 1 min, after which the trial was stopped, and the hanging time was recorded as 1 min.

#### Horizontal Bar Test

The mouse was gently placed on a metal wire, with the two forepaws gripping the wire. The time spent hanging on the wire was recorded. The maximum hanging time was set to 1 min. The average hanging time was calculated from 5 consecutive trials.

#### Rotarod Test

Mice were habituated to the rotarod apparatus (Touch Screen Rotarod, Harvard Apparatus, Holliston, USA) by leaving them on the rod rotating at a low speed (4 r/min) for 5 min each day for 3 days and tested on day 4 on the accelerating rod. The time and the maximum rotation speed at which the test mouse maintained balance on the rod were measured. Each mouse completed five consecutive trials.

#### Social Preference Test

A modified three-chamber apparatus was used. The apparatus comprised 3 rectangular chambers (25 cm × 38 cm) made of white Plexiglas with a 13-cm gate connecting the two side chambers to the middle chamber. A 3-sided fence (13 cm wide for each side) made of transparent Plexiglas was placed inside each side-chamber facing the door of the side-chambers, creating a 13 cm × 13 cm square area separated from the side chambers but connected to the middle chamber through the door (Fig. 7A). The two side-chambers were covered by transparent Plexiglas to minimize odorant diffusion. The test mouse was placed inside the middle chamber and freely explored the middle chamber and the square zone in each side-chamber for 10 min. Three social partner mice were then placed into the fenced area in one side-chamber, and the test mouse was allowed to explore freely for another 10 min. Another 3 social partner mice were then placed in the other side-chamber, and the behavior of the test mouse was tracked for 10 min. The time that the test mouse spent in each chamber was measured.

### Experimental Design and Statistical Analysis

The effects of three different gene features on gene co-expression profiles were first analyzed. MGCA (Fig. 1) was then applied to determine the convergence in developmental expression profiles of hcASDs and detect genes that were significantly co-expressed with hcASDs in the whole genome. The effectiveness of MGCA in predicting putative ASD-associated genes was analyzed by comparing its results with those of GCA, which does not consider the effects of three gene features. *CDH11* and *CDH9* were selected as example genes, and behavioral experiments were conducted in gene-knockout mice to test the findings of MGCA.

Data are presented as the mean ± SEM. The upper fence test and Grubbs’ test were used to evaluate whether the hcASD–hcASD expression level (CEC value) was significantly higher than those of feature-matched (mRand–mRand; hcASD–mRand) or non-matched, non-hcASD gene sets (Rand–Rand; hcASD–Rand). Grubbs’ test was done using the “grubbs.test” script in the R software package. The FDR of a gene was determined by the frequency of this gene significantly co-expressed (*P* < 0.001) with 5,000 mRand gene sets determined by MGCA. The gene enrichment *P-*value was determined with the *χ*^2^-test. Pathway enrichment *P-*values were determined with Metascape. Behavioral data were analyzed by Student’s *t*-test and by one-way ANOVA followed by Dunnett’s *t*-test as *post hoc* analysis using SPSS (IBM, Armonk, USA) or GraphPad Prism (GraphPad Software, La Jolla, USA).

#### Availability of Data and Materials

Perl scripts for data analysis are available on GitHub (https://github.com/wunan124/MGCA).

## Results

### Effects of Gene Features on Gene Co-expression Profiles

**Fig. 2:**
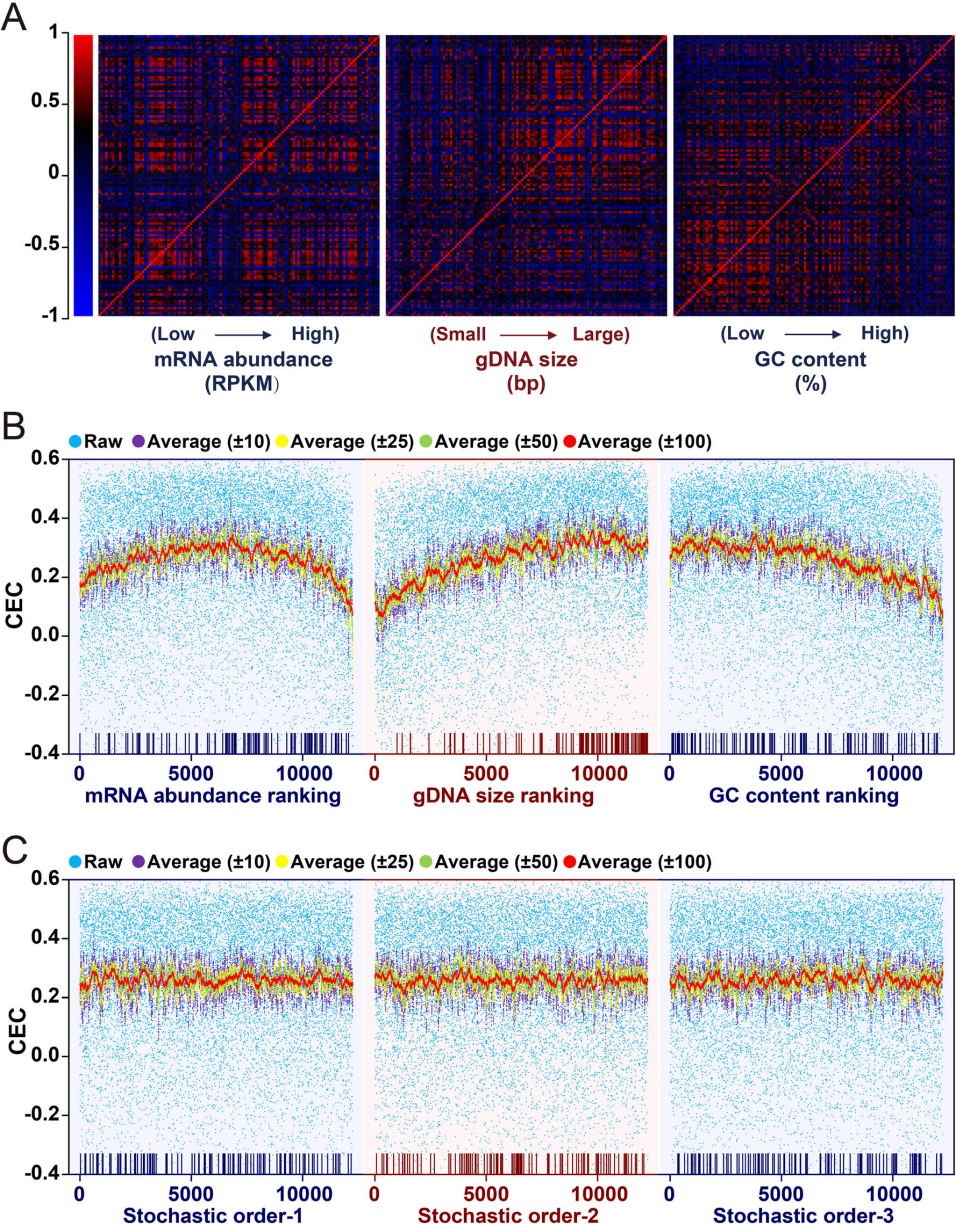
Effect of gene features on genome-wide gene co-expression profiles. A total of 12,250 genes with information on all 3 features were identified, placed in Table S1 as lists, and used for analyses. **A** Heatmaps of correlation coefficients (CCs) of genome-wide gene pairs. Genes are ranked according to mRNA abundance, gDNA size, or GC content. The CC of each gene with all genes is plotted and displayed in pseudo-color-coded matrices. **B** Genome-wide distribution of CECs of each gene with the hcASD gene set under three different gene ranking conditions. In each of the 3 matrix panels, the 12,250 genes are placed in ascending order on the *x*-axis, with 1 being the lowest mRNA abundance and GC content or shortest gDNA and 12,250 being the highest in mRNA abundance and GC content or the longest gDNA. Each blue dot represents the CEC of a gene with the hcASD gene set. Purple, yellow, green, and red dots represent the noise-reduced (average) CEC of a gene with its neighboring 20 (10 above and 10 below; ±10), 50 (±25), 100 (±50), or 200 (±100) genes on the lists. Rods at the bottom of each panel show the locations of hcASD genes on the ranked lists. **C** Genome-wide distribution of CECs of each gene with the hcASD gene set when they are placed in stochastic (random) order.

The potential effect of the three gene features – mRNA abundance, genomic DNA (gDNA) size, and GC content – on gene co-expression profiles was first analyzed using the BrainSpan human brain transcriptome dataset. This dataset contains the transcriptomes of human (both sexes) brain tissues from 16 different regions at various developmental stages and ages (from PCW8 to 40Y). A total of 12,250 genes with information on all 3 features were placed in ascending order of mRNA abundance, gDNA size, or GC content as ranked gene lists (Table S1). The CC of each gene pair was calculated to reflect the co-expression level of the two genes, and the results were displayed in pseudo-color-coded matrices. In each of the CC matrices (Fig. 2A), these 12,250 genes were placed in ascending order on both *x* and *y* axes. All three CC matrices exhibited variable color intensity in different areas with higher intensity corresponding to higher CC values. The overall color intensity was the highest in areas corresponding to medium mRNA abundance, medium-to-large gDNA size, and low GC content (Fig. 2A). This result suggests that all three gene features affect gene co-expression profiles.

Most hcASDs are large genes with medium-to-large mRNA abundance but with no apparent bias in GC content (Fig. S1). To determine whether each of these three gene features affects the co-expression of a gene with the hcASD gene set as a whole, the CEC (mean CC between a gene and each of the hcASD genes) of each of the 12,250 genes with the entire hcASD gene set was calculated (blue dots in Fig. 2B; Table S1). In each of the 3 panels (Fig. 2B), the 12,250 genes were placed in ascending order (*x*-axis). A noise-reduced (by data averaging) CEC distribution curve was then generated by plotting the average CEC of a gene with its neighboring 20 (10 above and 10 below; ±10), 50 (±25), 100 (±50), or 200 (±100) genes in the lists under each ranking condition. The results showed a bell-shaped curve when genes were ranked by mRNA abundance, suggesting that genes with medium expression levels are more likely to be co-expressed with the hcASD gene set (Fig. 2B, left panel). There was an overall positive correlation between the gDNA size of a gene and its CEC with the hcASD gene set (Fig. 2B, middle panel). The CEC maintained a relatively high level (0.28–0.35) when the GC content ranged from low to medium (approximately <45%, *x*-axis <6000) and then gradually declined with increasing GC content (Fig. 2B, right panel; Table S1).

With cubic regression, each noise-reduced CEC distribution curve was found to have an *R*^2^-value >0.88 (Fig. S2; Table S2), indicating a significant correlation between each of these gene features and the tendency for co-expression of a gene with the hcASD gene set. When the 12,250 genes were placed in stochastic (random) order, CECs were evenly distributed, and the noise-reduced CEC distribution curves were largely flat (Fig. 2C).

Similar genome-wide gene co-expression profiles of the hcASD gene set were found in the transcriptomes of early (PCW8 to 2Y) and late (4Y–40Y) stages (Fig. S3A), both sexes, and different brain regions (Fig. S3B, C). These findings suggest that the co-expression profile of hcASD genes is affected by all three gene features, regardless of developmental stage, sex, and brain area. Similar effects of these three features on the co-expression profiles of hcASD genes were found when a set of 64 high-susceptibility genes [24] were used as the hcASD gene set (Fig. S4).

### Similar Co-expression Profiles of Feature-matched Gene Sets

**Fig. 3:**
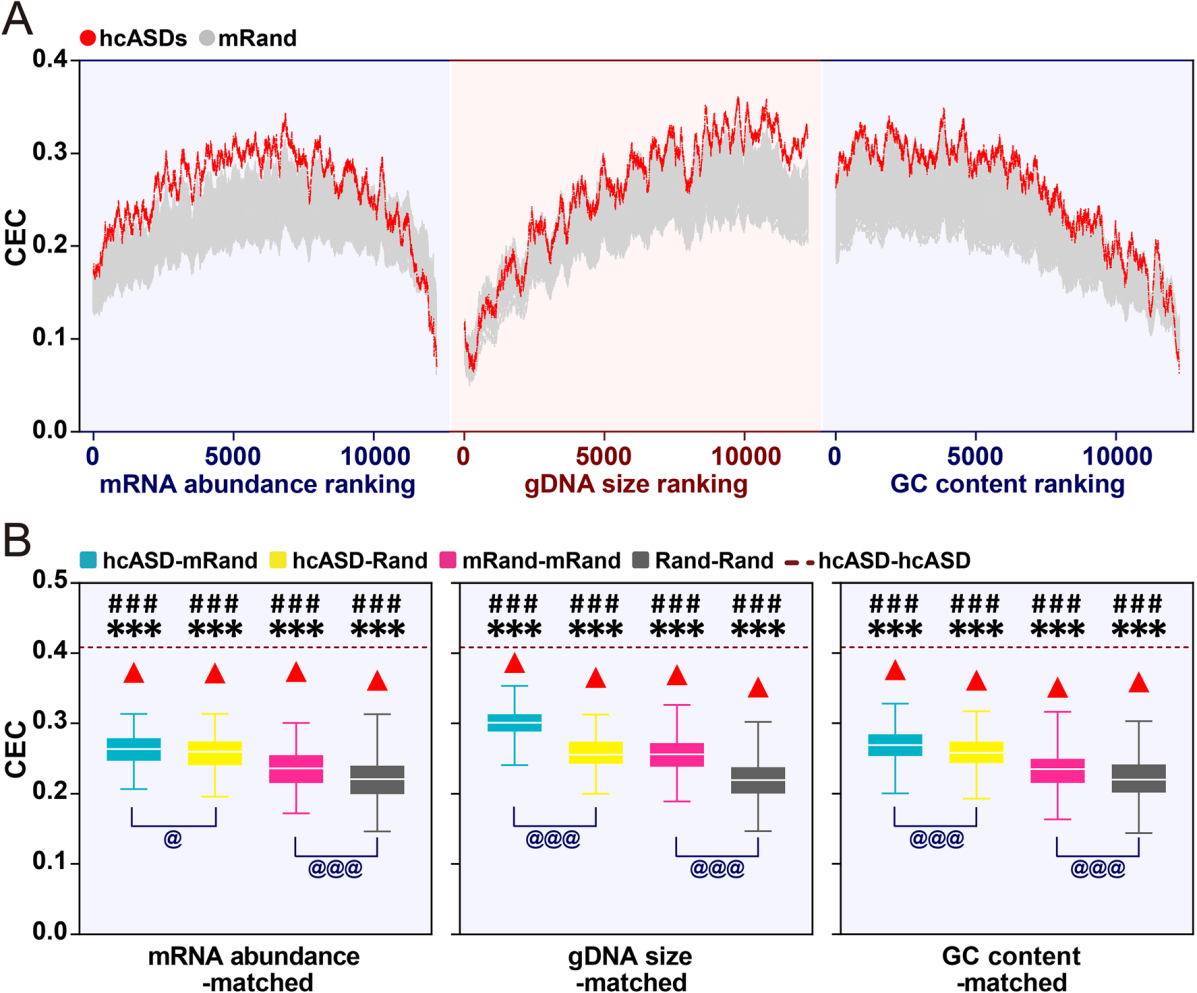
Convergent expression of hcASD genes determined by MGCA. **A** Comparison of noise-reduced CEC distribution curves between the hcASD gene set and 200 matched random gene sets (mRand) under different gene-ranking conditions. The *x*-axis represents gene rank. **B** CECs of hcASD–hcASD, hcASD–mRand, mRand–mRand, hcASD–Rand, and Rand–Rand gene set pairs. Two hundred each of mRand and Rand gene sets were analyzed. Box plots show ranges of CECs of hcASD–mRand, mRand–mRand, hcASD–Rand, and Rand–Rand gene set pairs. In each box plot, the central rectangles span the first quartile to the third quartile of 200 ranked CEC values. The white bar inside the rectangle shows the median CEC value, and whiskers above and below the box show the maximum and minimum values, respectively. The dotted line represents the CEC among hcASDs (hcASD–hcASD) in each panel. Three statistical methods are used to determine whether the CEC of hcASD–hcASD is significantly higher than that of hcASD–mRand, mRand–mRand, hcASD–Rand, and Rand–Rand. Upper fence test: red triangles stand for the boundaries of significant difference (3× fences). Grubbs’ test: ***P < 0.001. Permutation test: ^###^*P* < 0.001. Student’s *t*-test was used to determine whether the CECs of hcASD–mRand and mRand–mRand are significantly greater than those of hcASD–Rand and Rand–Rand, respectively. ^@^*P* < 0.05; ^@@@^*P* < 0.001.

The genome-wide gene co-expression profile of the hcASD gene set was then compared to the profiles of 200 feature-matched non-hcASD gene sets. Each gene set comprised an equal number (101) of randomly-selected and feature-matched non-hcASD genes under the three different gene ranking conditions (Fig. 3A). These gene sets were named “matched random” (mRand) sets (see Methods). In general, the genome-wide CEC distribution of hcASDs was similar to that of each of the 200 mRand sets under all three gene-ranking conditions. These findings suggest that gene sets with matched gene features have a genome-wide co-expression profile similar to the hcASD gene set. However, genes with low to moderate mRNA abundance (~1.2–30 RPKM, 1–10500 on the *x*-axis) had higher noise-reduced CECs with the hcASD gene set than with any of the 200 mRand gene sets. In contrast, genes with high mRNA abundance (>30 RPKM; 10500–12250 on the *x*-axis) had lower noise-reduced CECs with the hcASD gene set than with most mRand gene sets. Moreover, medium-to-large genes had higher noise-reduced CECs with the hcASD gene set than with most size-matched mRand gene sets. Apart from those with the highest GC content, most genes had higher noise-reduced CECs with the hcASD gene set than with most GC content-matched mRand gene sets.

### Co-expression of ASD Risk Genes

To determine whether hcASDs exhibit a significant tendency for co-expression with each other, the mean CEC of each of the 101 hcASDs with the hcASD gene set as a whole (hcASD–hcASD, see Methods) was compared to that of a large number of permuted gene sets, each comprising an equal number of feature-matched non-hcASD genes (mRand–mRand) or randomly-selected non-hcASD genes (Rand–Rand), and to the CEC between hcASD and mRand (hcASD–mRand) or Rand (hcASD–Rand) gene sets. Two hundred each of mRand and Rand gene sets were first analyzed, and the results showed that feature-matched gene sets (mRand) had overall higher CECs than random gene sets (Rand) under all three matched conditions (^@@@^ in Fig. 3B), suggesting that genes with similar features tend to co-express with each other. The CEC of hcASD–hcASD (dashed line in Fig. 3B) was beyond the 3 times interquartile range [Q3 + 3 × (Q3–Q1), 3× upper fence] of the CECs of mRand–mRand, Rand–Rand, hcASD–mRand, and hcASD–Rand gene sets. The results of Grubbs’ test confirmed this tendency (*** in Fig. 3B). These results suggest that hcASDs have a significantly greater tendency for co-expression with each other than with other feature-matched non-hcASD genes or randomly-selected genes. To corroborate this finding, a permutation test was conducted with 100,000 permuted sets of genes with matched or non-matched features. The CEC of hcASD–hcASD was again found to be significantly larger (permutation *P* < 0.00001) than that of hcASD–mRand, mRand–mRand, hcASD–Rand, or Rand–Rand (^###^ in Fig. 3B), indicating a significant co-expression tendency of hcASDs.

Significant co-expression of hcASDs was also found in transcriptomes of brain tissue at both early (PCW8 to 2Y) and late (4Y–40Y) stages (Fig. S5A, B), both sexes (Fig. S5C, D), and different brain regions (Fig. S6A–C). These results indicate a highly conserved co-expression profile of hcASDs. Combined ranking of −log_10_(*P-*values) of Grubbs’ test under all three matched conditions was then performed to determine the relative significance level of co-expression of hcASDs with each other in different brain regions (Fig. S6D). The top four regions with the highest significance levels were the cerebellum (CB), striatum (STR), orbital frontal cortex, and dorsal frontal cortex; these are regions previously implicated in ASD [25–32]. These results suggest that hcASDs play important roles in the development and function of these ASD-relevant brain regions.

### ASD-associated Genes and Pathways Identified by MGCA

**Fig. 4:**
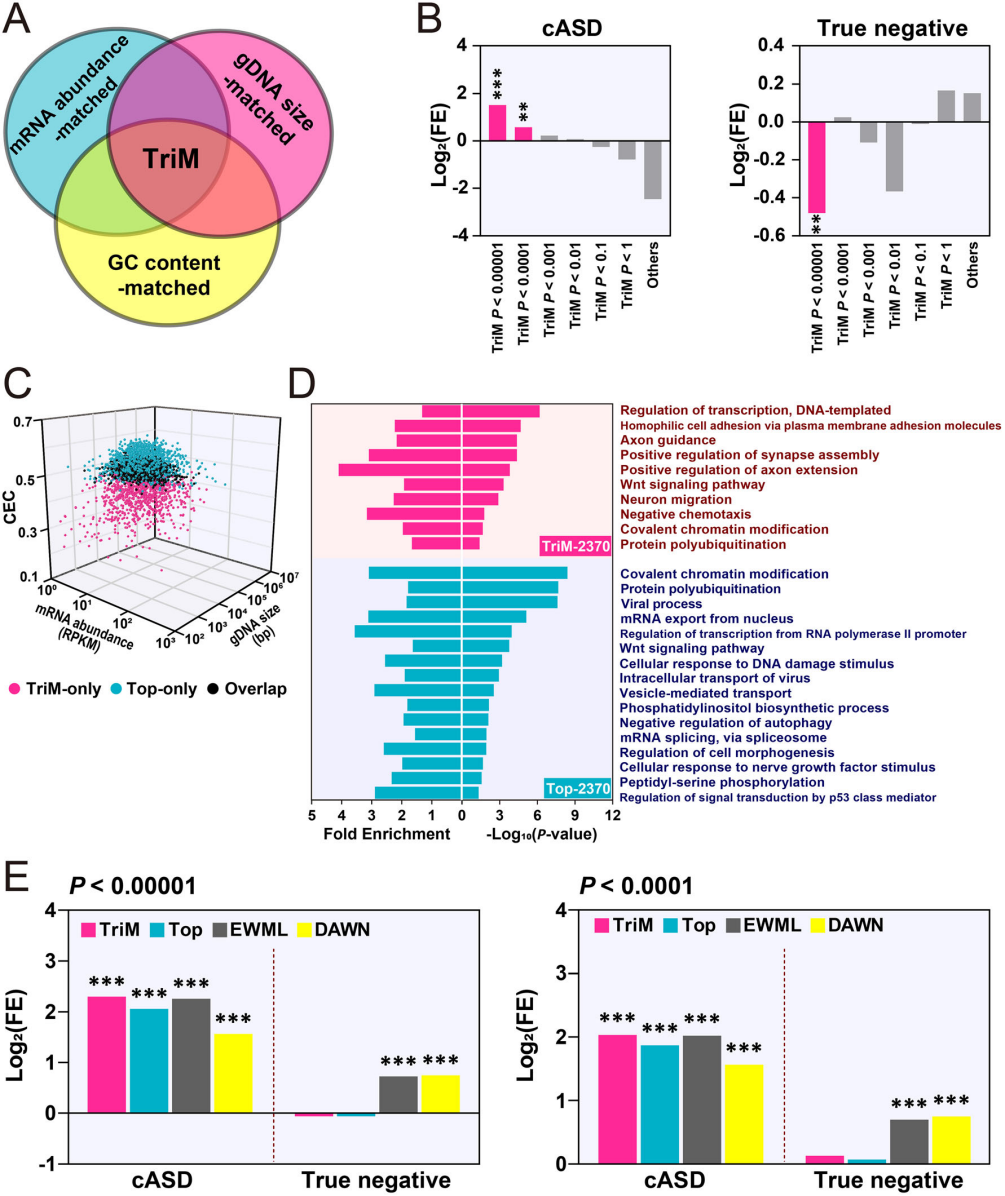
Evaluation of TriM genes identified by MGCA. **A** Schematic of TriM gene identification. **B** Fold enrichment (log_2_) of cASD (left) and “True negative” (right) genes in different groups of TriM genes (**P < 0.01, ***P < 0.001, *χ*^2^-test). **C** Three-dimensional distribution of TriM genes (*P* < 0.0001) and an equal number (2370) of genes with the highest CEC with hcASDs (top). The three axes are CEC value, gDNA size (bp), and mRNA abundance (RPKM). Each dot represents a gene (purple, TriM-specific; blue, Top-specific; black, overlapped). **D** Gene Ontology (GO) analysis of TriM (*P* < 0.0001) and Top (2370) genes showing significantly enriched GO terms (*P* < 0.05, Benjamini–Hochberg correction). **E** Comparison of the enrichment of cASD genes and “True negative” genes in TriM, Top, EWML-identified, and DAWN-identified genes (***P < 0.001, *χ*^2^-test).

Genes for which the CECs with hcASDs were significantly higher than with permuted gene sets composed of feature-matched genes under each of the three matched conditions were considered to be significantly co-expressed with hcASDs (estimated FDR of each gene <1.25 × 10^−4^). These were named TriM (triple-matched) genes (Figs 1, 4A; Table S3). TriM genes were then compared with a gene set containing 514 non-redundant genes from nine different sets of previously reported ASD-associated genes (cASD, see Methods) and with a set of “True negative” ASD-associated genes, which were associated with non-mental health diseases and not with ASD [12]. When the permutation *P-*value was either <0.0001 or <0.00001, the TriM set showed a significant enrichment of cASD genes (*P* = 0.0027 and *P* < 0.0001, respectively, *χ*^2^-test; Fig. 4B). This result suggests that at a permutation *P-*value < 0.0001, TriM genes have a high rate of positive prediction of being ASD-associated genes. When the permutation *P-*value was < 0.00001, TriM genes exhibited a significant negative enrichment of “True negative” genes (*P* = 0.0068, *χ*^2^-test; Fig. 4B), suggesting a significantly low false-positive rate in the prediction of ASD-associated genes.

Altogether, MGCA revealed 2370 TriM genes with a permutation *P-*value < 0.0001. These genes (TriM-2370) were compared with an equal number (2370) of genes that had the highest CECs with the hcASD gene set (referred to as the Top-2370 gene set, Table S3). The TriM-2370 and Top-2370 gene sets had 1414 genes in common (overlapped), and each had 956 non-overlapped genes. These two non-overlapped gene sets were named TriM-only and Top-only, respectively (Fig. 4C; Table S3). Most Top-only genes had a medium mRNA abundance, a large gene size, and a high CEC value (>0.46), whereas TriM-only genes had a broad range of mRNA abundance, gene size, and CEC values (Fig. 4C). GO enrichment analysis of the TriM-2370 gene set showed significant over-representation of genes in molecular pathways closely related to ASD: gene transcription regulation, homophilic cell adhesion, axon guidance and axon extension, synapse assembly, Wnt signaling pathway, neuron migration, covalent chromatin modification, and protein polyubiquitination. Fewer pathways relevant to ASD were revealed in the Top-2370 gene set by GO analysis: covalent chromatin modification, transcription regulation, protein polyubiquitination, Wnt signaling pathway, and negative regulation of autophagy (Fig. 4D; Table S4). To investigate the functional relationship between cASD and TriM-2370 or Top-2370 gene sets, the enriched molecular pathways of cASD, TriM-only, Top-only, and overlapped gene sets were subjected to pathway enrichment analysis [33] (Fig. S7). The results showed that the TriM-only and Top-only sets converged on different but complementary molecular pathways. The molecular pathways of the cASD gene set were found to cluster closer to those of the TriM-only set than to those of the Top-only set, suggesting that TriM-only genes have a closer functional relationship with cASD genes than with Top-only genes. Some well-established ASD risk genes, such as *FOXP1*, *TBR1*, *SHANK2*, *SYNGAP1*, and *PCDH9*, were found in the TriM-only gene set, suggesting a better performance of MGCA than conventional GCA in revealing molecular pathways relevant to ASD.

The effectiveness of MGCA in predicting genes relevant to ASD was compared with that of GCA and two other risk gene prediction algorithms. One was DAWN (detecting association with networks), which has been used to analyze the association between rare genetic variations and gene co-expression in the mid-fetal prefrontal and somatosensory cortex [34]. The other algorithm was EWML (evidence-weighted machine learning) that has been used to predict the probability of ASD association with whole-genome genes based on data from gene co-expression, genetic mutations, and protein-protein interactions [12]. The combined ASD risk gene set (cASD) and the “True negative” gene set were used to conduct cross-comparisons between the different algorithms. At a permutation *P-*value of 0.0001 or 0.00001, TriM genes had higher enrichment of cASD genes than an equal number of genes with the highest CEC values (Top), an equal number of top-ranked ASD-linked genes predicted by EWML, and network ASD genes identified by DAWN (Fig. 4E). Furthermore, fewer TriM genes overlapped with “True negative” genes than those predicted by DAWN and EWML, which had significant enrichment of “True negative” genes (Fig. 4E), suggesting a lower rate of false-positive prediction by MGCA than that by EWML and DAWN (Fig. 4E). Thus, MGCA performs better than GCA by a higher positive prediction rate and performs better than the EWML and DAWN algorithms by both a higher positive prediction rate and a lower prediction error.

### Co-expression of Cadherin Genes with hcASDs

Consistent with previous findings [8], we found that homophilic cell adhesion is the most significantly over-represented pathway of TriM-2370 genes (Fig. 4D; Table S4). Some cadherin family members in the TriM-2370 gene set, such as *PCDH19*, are known to be high-risk ASD genes (Table S6) that play important roles in brain circuit development [35, 36]. Several cadherin family members were also found in the TriM-2370 gene set, including many members of the protocadherin β gene cluster and dachsous cadherin-related 1 (*DCHS1*), suggesting that these genes also participate in the development and function of brain circuits relevant to ASD. Some cadherin genes were not significantly co-expressed with hcASDs under any of the matched conditions; these genes were referred to as tri-negative genes (TriN; Table S6). Several recent genetic studies have implicated two type II cadherins, *CDH11* and *CDH9*, in ASD and other psychiatric diseases [37–41]. The CEC-values of *CDH11* and *CDH9* with hcASDs were ranked at 5244 and 9581, respectively, among whole-genome genes. Therefore, neither of them belonged to the top-ranked genes based on traditional GCA. Using MGCA, we found that *CDH11* and *CDH9* belonged to the TriM and TriN gene sets, respectively. We thus hypothesized that *CDH11*, but not *CDH9*, is more likely to be associated with ASD.

### Autism-like Traits of *Cdh11*-null Mice

**Fig. 5:**
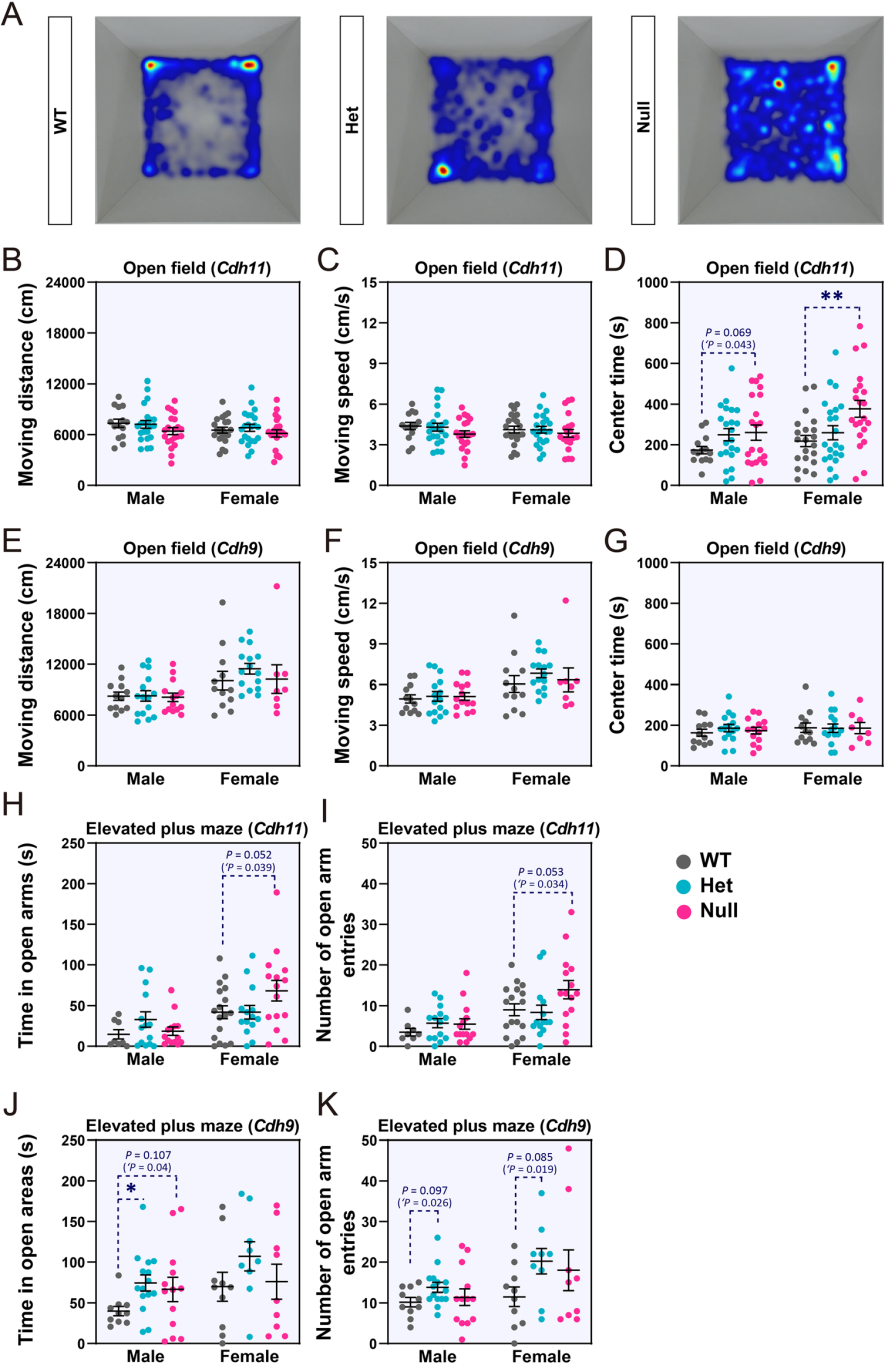
Open field and elevated plus maze tests of *Cdh11*- and *Cdh9*-null mice. **A** Heatmaps showing cumulative frequency of locations visited by *Cdh11*-null, heterozygotic (Het), and WT mice in the open field arena. **B–G** Distance moved, velocity, and center exploration time of *Cdh11*-null mice (**B**–**D**) and *Cdh9*-null mice (**E**–**G**) (male *Cdh11*-null: *n* = 21, Het: *n* = 22, WT: *n* = 14; female *Cdh11-*null: *n* = 21, Het: *n* = 22, WT: *n* = 21; male *Cdh9*-null: *n* = 14, Het: *n* = 15, WT: *n* = 12; female *Cdh9*-null: *n* = 8, Het: *n* = 15, WT: *n* = 12). **H**–**K** Time spent in open arms and open arm entries of *Cdh11*-null (**H**, **I**) and *Cdh9*-null (**J**, **K**) mice (male *Cdh11*-null: *n* = 14, Het: *n* = 14, WT: *n* = 8; female *Cdh11*-null: *n* = 15, Het *n* = 14, WT *n* = 17; male *Cdh9*-null: *n* = 13, Het: *n* = 15, WT: *n* = 10; female *Cdh9*-null: *n* = 9, Het: *n* = 9, WT: *n* = 10). Data are the mean ± SEM; ‘*P* < 0.05, Student’s *t*-test; *P < 0.05, **P < 0.01, one-way ANOVA followed by Dunnett’s *t*-test.

To assess the functional relevance of *CDH11* and *CDH9* to ASD, we investigated the behaviors of *Cdh11*- and *Cdh9*-null mice. In the open field test, both male and female *Cdh11*-null mice spent more time exploring the central area of the open field arena than WT littermates (Fig. 5A, D). Heterozygous littermates showed a similar but less significant pattern. Total distance moved and average velocity of *Cdh11*-null mice were slightly lower than in WT littermates (Fig. 5B, C). Both male and female *Cdh9*-null mice were largely normal in this test (Fig. 5E–G).

In the elevated plus maze test, female *Cdh11*-null mice visited the open arms more frequently and spent a significantly longer time there. Heterozygous females spent slightly but not statistically significantly more time in the open arms (Fig. 5H, I). The increased time and frequency of open arm exploration by female *Cdh11*-null mice is consistent with the results of a previous study using the same mouse line of mixed sex [42]. Male *Cdh9*-null mice showed longer exploration of the open arms, but female *Cdh9*-null mice did not, although female heterozygotes showed an increased frequency of open arm entry (Fig. 5J, K).

**Fig. 6:**
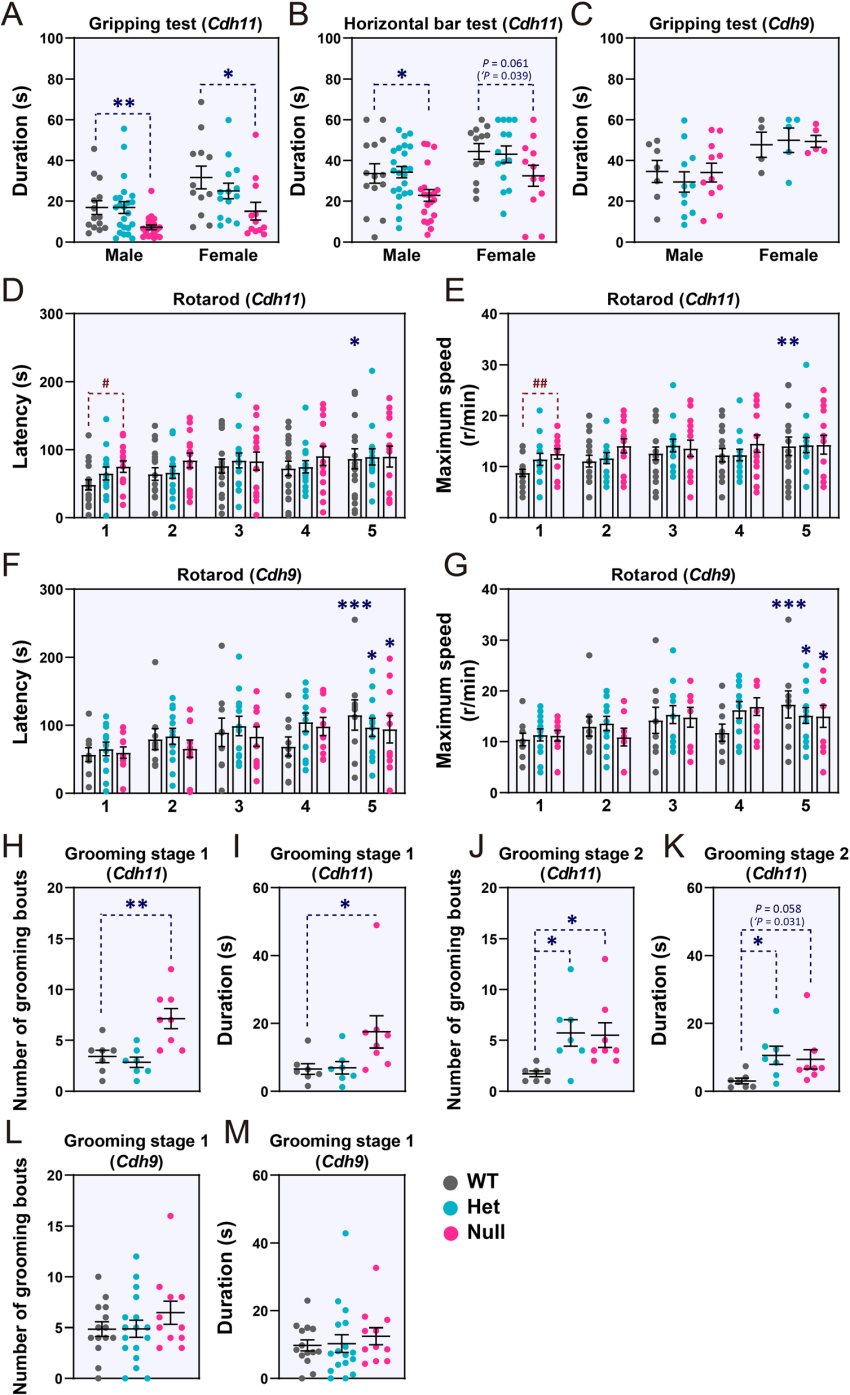
Grip-strength and repetitive behaviors of *Cdh11*- and *Cdh9*-null mice. **A, B** Results of grip test and horizontal bar test for *Cdh11*-null mice (male *Cdh11*-null: *n* = 21, Het: *n* = 23, WT: *n* = 14; female *Cdh11*-null: *n* = 12, Het: *n* = 14, WT: *n* = 12). **C,** Results of grip test for *Cdh9*-null mice (male *Cdh9*-null: *n* = 11, Het: *n* = 11, WT: *n* = 7, female *Cdh9*-null: *n* = 5, Het: *n* = 5, WT mice *n* = 4). **D–G** Latency to fall (**D, F**) and maximum speed (**E, G**) in the rotarod test for female *Cdh11*- and *Cdh9*-null mice (*Cdh11*-null: *n* = 14, Het: *n* = 14, WT: *n* = 17; *Cdh9*-null: *n* = 10, Het: *n* = 12; WT: *n* = 9). Numbers below the *x*-axis (1–5) represent different trials of tests. **H–K** Frequency and duration of self-grooming of female *Cdh11*-null mice during the first (stage 1, **H, I**) and the second (stage 2, **J, K**) 10 min in the open field arena (*Cdh11*-null: *n* = 8, Het: *n* = 7, WT *n* = 7). **L, M** Frequency and duration of self-grooming of female *Cdh9*-null mice during the first (stage 1) and second (stage 2) 10 min in the open field arena (*Cdh9*-null: *n* = 11, Het: *n* = 17, WT: *n* = 14). Data are the mean ± SEM; ‘*P* < 0.05, Student’s *t*-test; *P < 0.05, **P < 0.01, ***P < 0.001 *vs* first trial; ^#^*P* < 0.05, ^##^*P* < 0.01 *vs* WT littermates, one-way ANOVA followed by Dunnett’s *t*-test.

Individuals with ASD often have a weaker grip-strength than age-matched controls [43]. The grip-strength test and the horizontal bar test showed that both male and female *Cdh11*-null mice exhibited significantly shorter hanging duration than WT littermates (Fig. 6A, B), indicating a reduced grip-strength or impaired motor coordination. The grip-strength of *Cdh9*-null mice was normal (Fig. 6C).

The rotarod test was conducted to evaluate motor-related functions of null mice. Since female and male mutant mice displayed similar behaviors in most of the above behavioral tests, only female mice were analyzed in this test. Compared to WT littermates, *Cdh11*-null mice, but not *Cdh9*-null mice, stayed longer on the rotarod and endured a higher rotation speed in the initial trial (Fig. 6D–G). In subsequent trials, *Cdh11*-null mice did not display significant performance improvement (Fig. 6D, E), indicating impaired motor learning. The enhanced performance of *Cdh11*-null mice in the initial trial was similar to the phenotype of several other well-characterized ASD mouse models and suggested increased repetitive motion of these mutant mice [44].

Repetitive behaviors were then evaluated by measuring the duration and frequency of self-grooming within 10 min, during which mice were placed in a novel or a relatively familiar environment. During the first 10 min of exploring a novel chamber, *Cdh11*-null mice exhibited a significantly greater frequency of self-grooming than WT littermates, indicating elevated repetitive behavior in a novel environment (Fig. 6H, I). *Cdh11*-null mice also showed a significantly higher frequency of self-grooming than WT littermates during the second 10-min period (Fig. 6J, K), indicating elevated repetitive behavior even in a relatively familiar environment. No such behavioral alteration was observed in *Cdh9*-null mice (Fig. 6L, M).

**Fig. 7:**
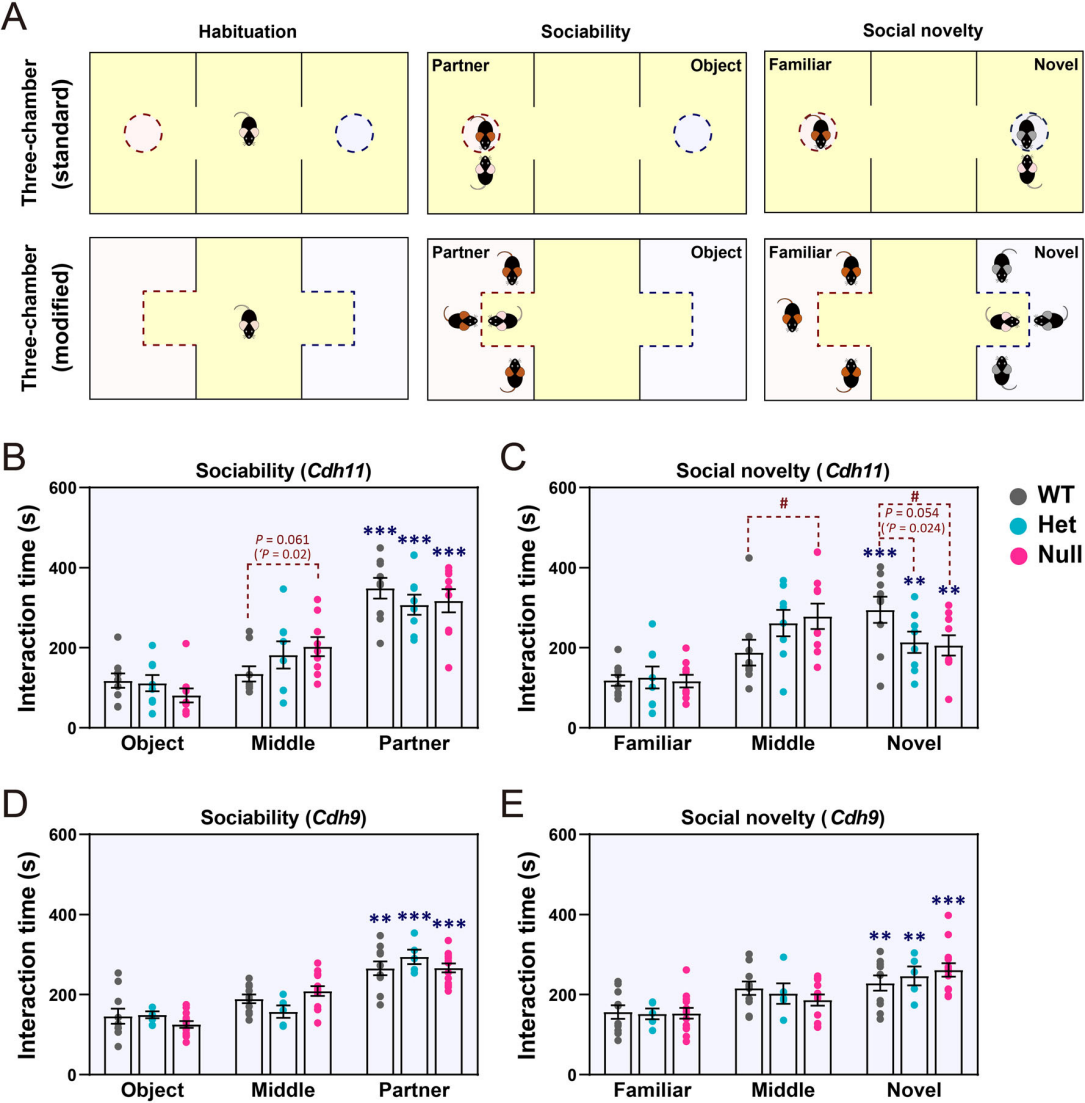
Modified three-chamber test of female *Cdh11*- and *Cdh9*-null mice. **A** Schematics of standard and modified three-chamber tests. **B**–**E** Results of sociability and social novelty preference tests of *Cdh11*-null (**B**, **C**; *Cdh11*-null: *n* = 9, Het: *n* = 8; WT: *n* = 9) and *Cdh9*-null mice (**D**, **E**; *Cdh9*-null: *n* = 13, Het: *n* = 5, WT: *n* = 10). Data are the mean ± SEM; ‘*P* < 0.05, Student’s *t*-test; *P < 0.05, **P < 0.01, ***P < 0.001 *vs* time spent on the other side of the chamber; ^#^*P* < 0.05 *vs* WT littermates, one-way ANOVA followed by Dunnett’s *t*-test.

The modified three-chamber social preference test was conducted to evaluate the sociability of mutant mice. One main modification was an enlargement of the area for housing social partner mice to reduce their potential stress and anxiety. Another major modification to the protocol was using three mice instead of a single mouse as social partners. This was done to increase the availability of social cues and reduce the variability of test results caused by differences in the sociability of individual social partners (Fig. 7A). In addition, the top of the two side-chambers was covered to slow the diffusion and mixing of odorant cues. The results showed that female *Cdh11*-null mice exhibited a significant preference for social partner mice than for an object and a significant preference for novel partners than for familiar ones (Fig. 7B, C). However, compared to WT littermates, mutant mice spent a significantly longer time in the middle chamber but a significantly shorter time interacting with partner mice (Fig. 7B, C), indicating reduced sociability. In contrast, *Cdh9*-null mice did not show any abnormality in this test (Fig. 7D, E).

## Discussion

GCA is a powerful tool to find functionally convergent genes. Several previous GCA studies have considered the potential effect of gene size and GC content on the co-expression of ASD risk genes [7]. In the present study, we discovered that three gene features – mRNA abundance, gDNA size, and GC content – affected the genome-wide co-expression profiles in the brain. Although the mechanisms by which different features affect co-expression are unknown, our findings suggest the importance of considering the effect of these gene features in GCA. As an example of the potential influence of confounding gene features on GCA, genes that are stably expressed in the brain may have high CCs with each other. These randomly high CCs of high-abundance genes do not mean a real co-expression relationship of the genes of common molecular pathways. One possible influence of gene size on GCA is that large genes may have long noncoding regions that could be regulatory elements. Thus, compared to small genes, large genes may have more shared regulatory elements, which means a higher chance of being co-regulated by common transcription factors. Considering that most hcASD genes are large, they may have a higher tendency for co-expression with large genes. Genes with high GC content have greater mRNA stability and thus have a greater chance of co-existence with each other. However, that most hcASD genes have low-to-medium GC content (Fig. 2B) may explain the overall negative correlation between their GC content and their co-expression with hcASDs.

Almost all previous studies ignored these important confounding factors and just selected high-CC gene pairs to construct a gene co-expression network. Without correcting for the effect of these factors, the effectiveness of GCA would be compromised. Instead of setting a CC threshold for GCA as in most other studies, we screened for significant co-expression relationships by comparing the CEC of a gene with the hcASD gene set to that with permuted gene sets with matched gene features. Only genes that had a CEC with the hcASD gene set significantly higher than its CECs with permuted sets of feature-matched genes were considered to be co-expressed with hcASDs. This MGCA paradigm (Fig. 1) allowed the demonstration of significant co-expression of hcASDs and avoided the potential bias caused by an empirically determined threshold for the CC of gene pairs in GCA. Our results revealed that MGCA is more efficient in predicting gene association than the pre-existing methods DAWN, an algorism integrating genetic variants and gene co-expression data, and EWML, a sophisticated machine-learning algorithm with the integration of gene co-expression, gene mutation databases, and protein–protein interaction networks. We believe that the high performance of MGCA could be attributed to the correction of three confounding gene features in the determination of functionally relevant gene co-expression. Although correlations of these confounding features with the co-expression with hcASDs have been considered [7], the potential interference of these features on the construction of gene co-expression networks was not considered in previous studies. Therefore, MGCA will be an important complement to current gene association prediction algorithms (Fig. 1). As MGCA is based solely on gene co-expression data, future algorithms combining MGCA with genetic mutation data and machine learning will further improve its efficacy.

An important finding in this study is the plausible association of *CDH11* with ASD determined by MGCA. Cadherins have been shown to accumulate in synaptic junctions and regulate dendrite development and synapse maturation [45–48]. Several cadherin family members, such as some protocadherins in the *FAT* cadherin subfamily, have been implicated in ASD [49–58]. A genetic association study of a large cohort of ASD individuals and matched controls revealed genes in the protocadherin-α cluster (*PCDHA*) to be ASD risk genes [51]. Mutations in the *PCDH19* gene have been shown to cause early-onset epilepsy, and many individuals with these mutations also display autistic features [52–54]. Mutations in the cadherin epidermal growth factor laminin G seven-pass G-type receptor 2 gene (*CELSR2*) are thought to be responsible for Joubert syndrome, a disease with a high degree of autistic features [59, 60]. It is uncertain whether other cadherins are also high-risk factors. Using MGCA, we found that a group of cadherin superfamily members exhibited high co-expression with hcASDs, suggesting shared functions with hcASDs and a role in ASD etiology. Among them, several protocadherins, mainly *PCDHB*s, exhibited significant co-expression with hcASDs (Table S6). The functions of these putative ASD-associated cadherins in the brain remain to be determined. One such cadherin identified by MGCA was CDH11. In this study, we found that *Cdh11*-null mice had significantly increased repetitive behaviors. The neocortex, CB, and STR are known to be involved in the control of repetitive behaviors [61]. It is likely that cadherins, *Cdh11* in particular, play important roles in mediating synapse formation during the wiring of circuits in these brain areas. Consistent with this postulate, our recent work showed *Cdh11* expression in ASD-associated sub-regions in the CB of the developing mouse brain [62].

In human studies, partial deletion of *CDH11* has been reported in a sporadic case of non-syndromic ASD, mild intellectual disability, and attention deficit hyperactivity disorder (ADHD) [37]. A case-control association study revealed a high prevalence of the homozygous single nucleotide variant rs7187376C/C of *CDH11* in patients with ASD [37]. Several other coding variants of *CDH11* have also been discovered in individuals with ASD [37]. Behavioral changes that we have observed in *Cdh11*-null mice, including reduced anxiety, increased repetitive behavior, and reduced sociability, are highly consistent with the non-syndromic ASD case with partial deletion of *CDH11* [37]. This finding supports the notion that loss-of-function of a single gene, such as *CDH11*, is sufficient to cause major autism traits. Recessive mutations have been implicated in ASD, and bi-allelic disruption of recessive neurodevelopmental genes in ASD has been reported [62]. We found that homozygous, but not heterozygous *Cdh11*-null mice displayed autism-like behavioral deficits. This suggests that *CDH11* may be a recessive ASD gene [63]. However, clinical data from more families with *CDH11* mutations are needed to determine whether this is true. Behavioral phenotypes of ASD are highly heterogeneous. Some individuals with ASD are hypoactive with elevated anxiety, and some have ADHD but with reduced anxiety [64–67]. The genetic and neurobiological mechanisms underlying this behavioral heterogeneity have not been fully determined. Further investigation with a larger cohort of patient families is needed to determine whether loss-of-function mutations of *CDH11* are associated with ADHD.

Most genetic variants found in patients with ASD are heterozygous. In some behavioral tests, heterozygous *Cdh11*-null mice showed a trend of behavioral alterations similar to homozygous null mice, but not at a statistically significant level (Figs 6J, K, and 7C). As ASD has a complex genetic basis and is affected by environmental factors, it is conceivable that the haplodeficiency of a single risk gene causes a relatively mild behavioral phenotype in mice. More severe behavioral deficits may result if the haplodeficiency of *Cdh11* is combined with other genetic or environmental factors. Our findings suggest that *CDH11* is significantly co-expressed with hcASDs and that its mutations may have a causal effect on autism traits. *Cdh11*-null mice could be very useful in dissecting the circuit mechanisms underlying a subgroup of ASD and in screening drugs targeting this subgroup of ASD.

*CDH9* plays a vital role in establishing specific synaptic wiring in both the hippocampus and the retina [22, 68]. Its association with ASD has been suggested by several studies on exome sequencing [49, 69]. The primary evidence linking *CDH9* to ASD is the strong association of the single nucleotide polymorphism rs4307059 located in the intergenic region between *CDH10* and *CDH9* with ASD [70]. However, this rs4307059 genotype is not correlated with the expression of either *CDH9* or *CDH10* in the adult brain [70, 71], and whether a correlation exists in the fetal brain is unknown. Recently, an antisense noncoding RNA of moesin pseudogene 1 (MSNP1AS) was shown to be transcribed from the locus harboring rs4307059. Alterations in this pseudogene have been postulated to contribute to ASD [71–73]. Whether *CDH9* deficiency is a causal factor for ASD remains undetermined. Our MGCA showed that, unlike *CDH11*, *CDH9* was not co-expressed with hcASDs. This is an indication that *CDH9* may not play an essential role in the wiring of ASD-relevant circuits. Consistent with this notion, behavioral tests showed that *Cdh9*-null mice exhibited a very mild behavioral abnormality only in the elevated plus maze test but not in any other tests. With recent findings by other researchers [74], our results suggest that *CDH9* deficiency may not have a significant effect on autism traits.

In conclusion, this study revealed the importance of considering matched gene features in the analysis of gene co-expression and demonstrated the effectiveness of MGCA in the identification of putative ASD-associated genes and their convergent signaling pathways. The application of MGCA led to the determination of *CDH11* as a putative ASD-associated gene. Our results also showed that *Cdh11*-null mice can be used to study the circuit mechanisms of a subgroup of ASD and explore therapeutic strategies for ASD.

## Supplementary Information

Below is the link to the electronic supplementary material.

Supplementary file 1 (PDF 2867 KB)

Supplementary file 2 (XLS 5194 KB)

Supplementary file 3 (XLS 32 KB)

Supplementary file 4 (XLS 4802 KB)

Supplementary file 5 (XLS 344 KB)

Supplementary file 6 (XLS 655 KB)

Supplementary file 7 (XLS 37 KB)
