# Supplementary material for: Association of CDH11 with Autism Spectrum Disorder Revealed by Matched-gene Co-expression Analysis and Mouse Behavioral Studies
Source: Neurosci Bull. 2021 Sep 14;38(1):29–46. doi: 10.1007/s12264-021-00770-0 (PMC8783018; doi:10.1007/s12264-021-00770-0)
Supplement: Supplementary file 1 — (PDF 2867 KB) [file 12264_2021_770_MOESM1_ESM.pdf]

Supplementary Materials

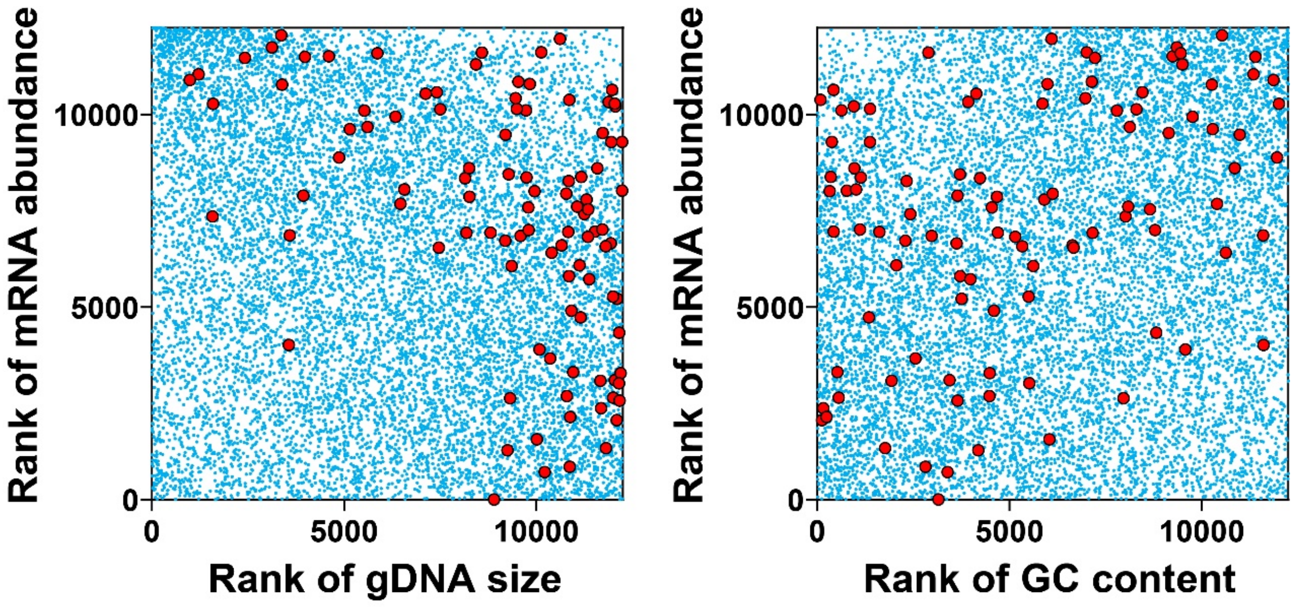

Fig. S1 Distribution of hcASDs in gene rank matrices. Genes in the whole genome are ranked by gDNA size, GC content (horizontal axis), and mRNA abundance level (vertical axis) and plotted in matrices (blue dots, single genes; red dots, hcASD genes). Values of both horizontal and vertical axes are ranks (orders) of the 12,250 genes under 3 different gene ranking conditions.

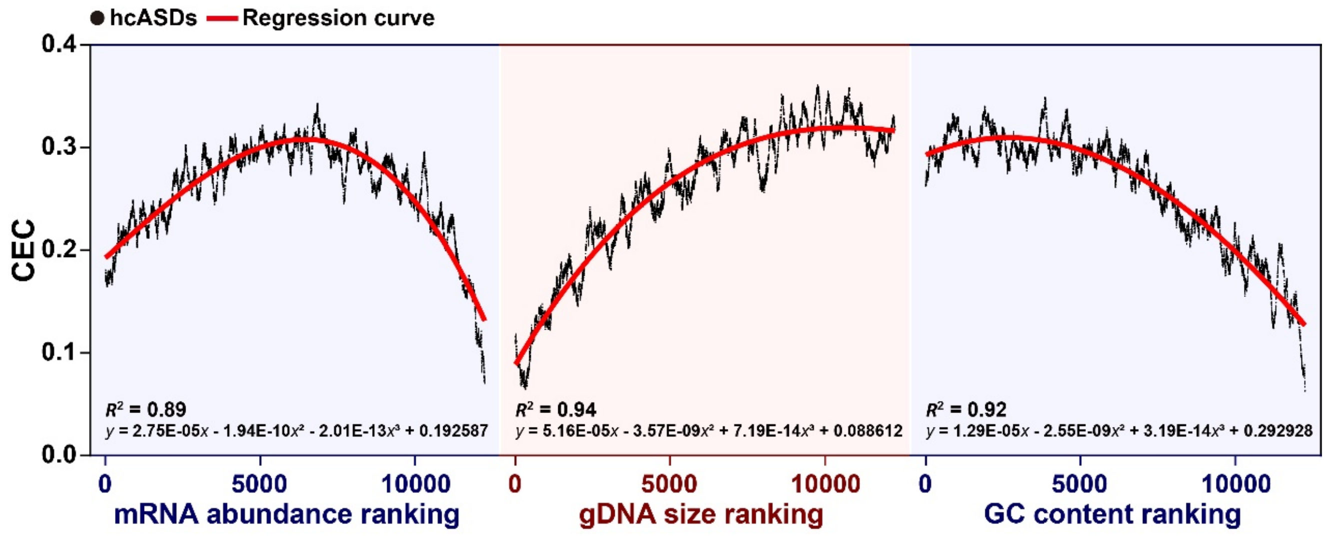

Fig. S2 Fitting (regression) of CEC distribution curves under three different gene ranking conditions.

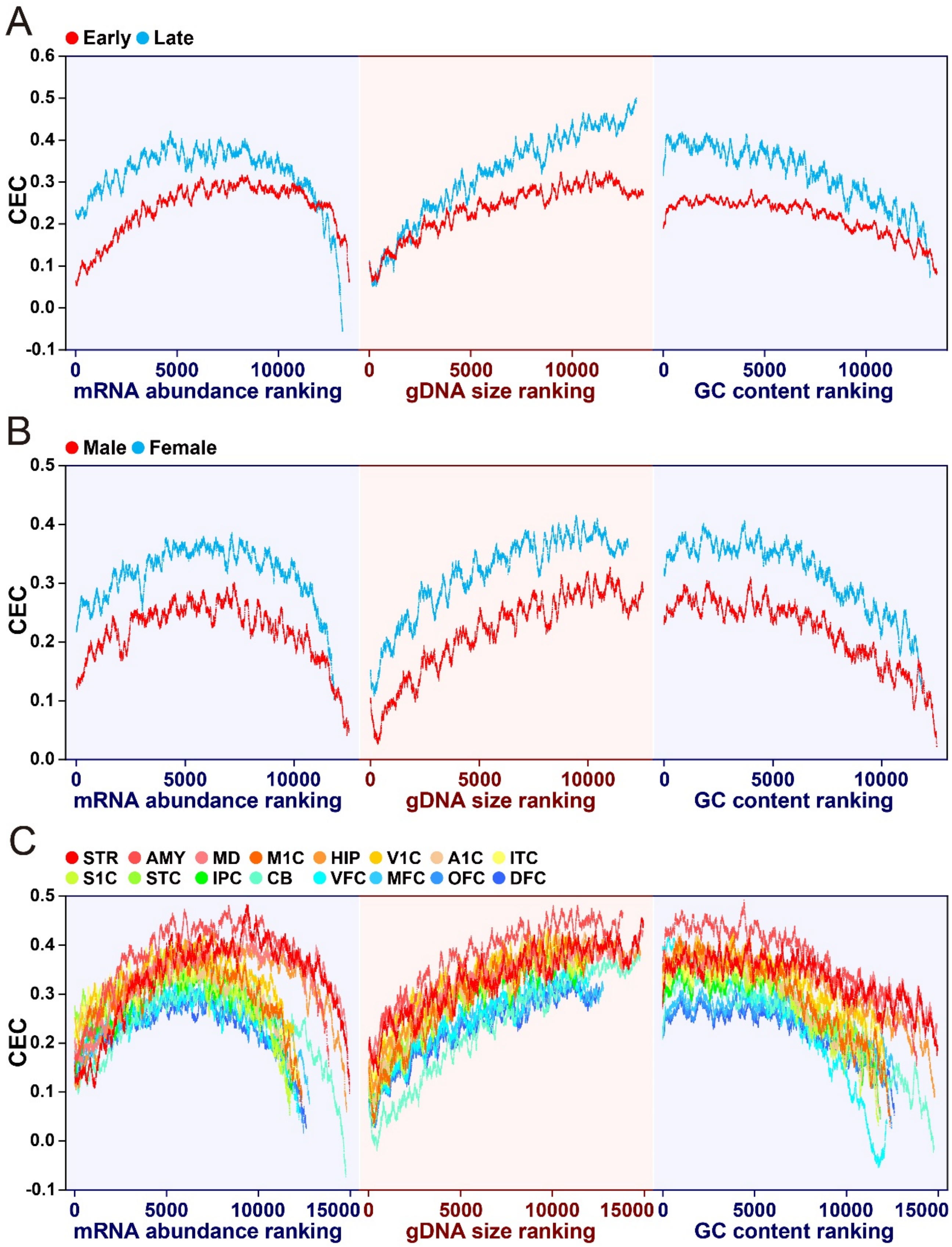

Fig. S3 CEC distribution curves for different developmental stages, sex, and brain regions.

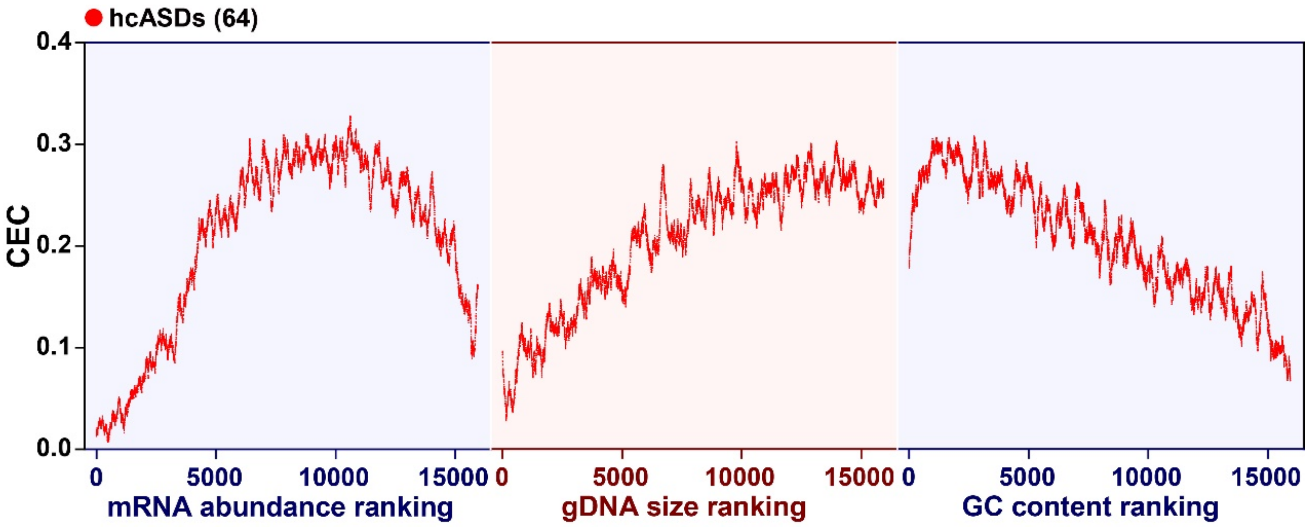

Fig. S4 Genome-wide distribution of CECs of each gene with the hcASD (64) gene set under three different gene ranking conditions.

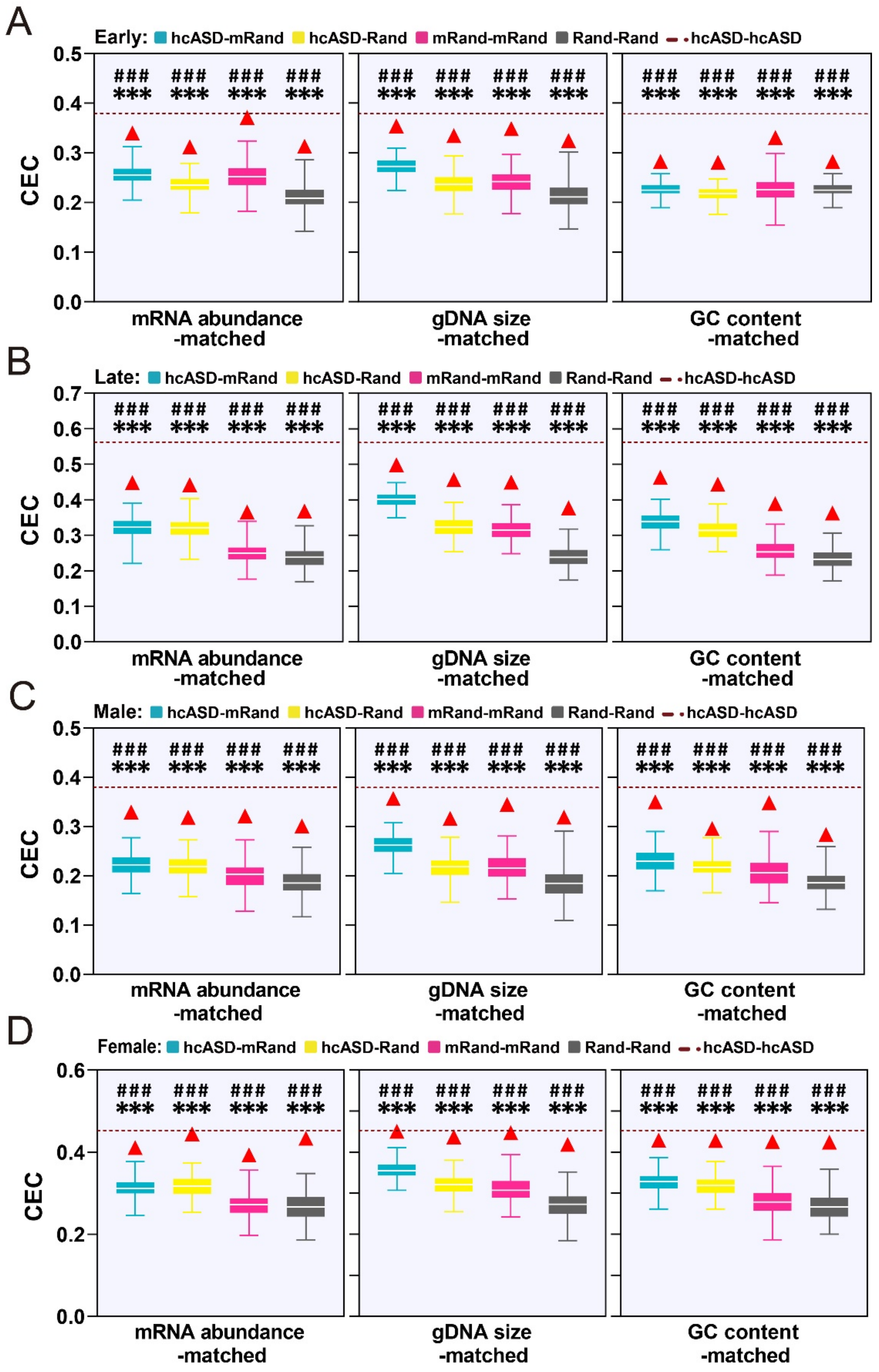
A
Early: hcASD-mRand hcASD-Rand mRand-mRand Rand-Rand hcASD-hcASD
CEC
mRNA abundance -matched
gDNA size -matched
GC content -matched
B
Late: hcASD-mRand hcASD-Rand mRand-mRand Rand-Rand hcASD-hcASD
C
Male: hcASD-mRand hcASD-Rand mRand-mRand Rand-Rand hcASD-hcASD
D
Female: hcASD-mRand hcASD-Rand mRand-mRand Rand-Rand hcASD-hcASD

Fig. S5 Co-expression of hcASDs in the brain at different developmental stages and sex. **A**, **B** Data from early (**A**; PCW8 to 2Y) and late (**B**; 3Y–40Y) developmental stages. **C**, **D** Data from male (**C**) and female (**D**) brain tissue.

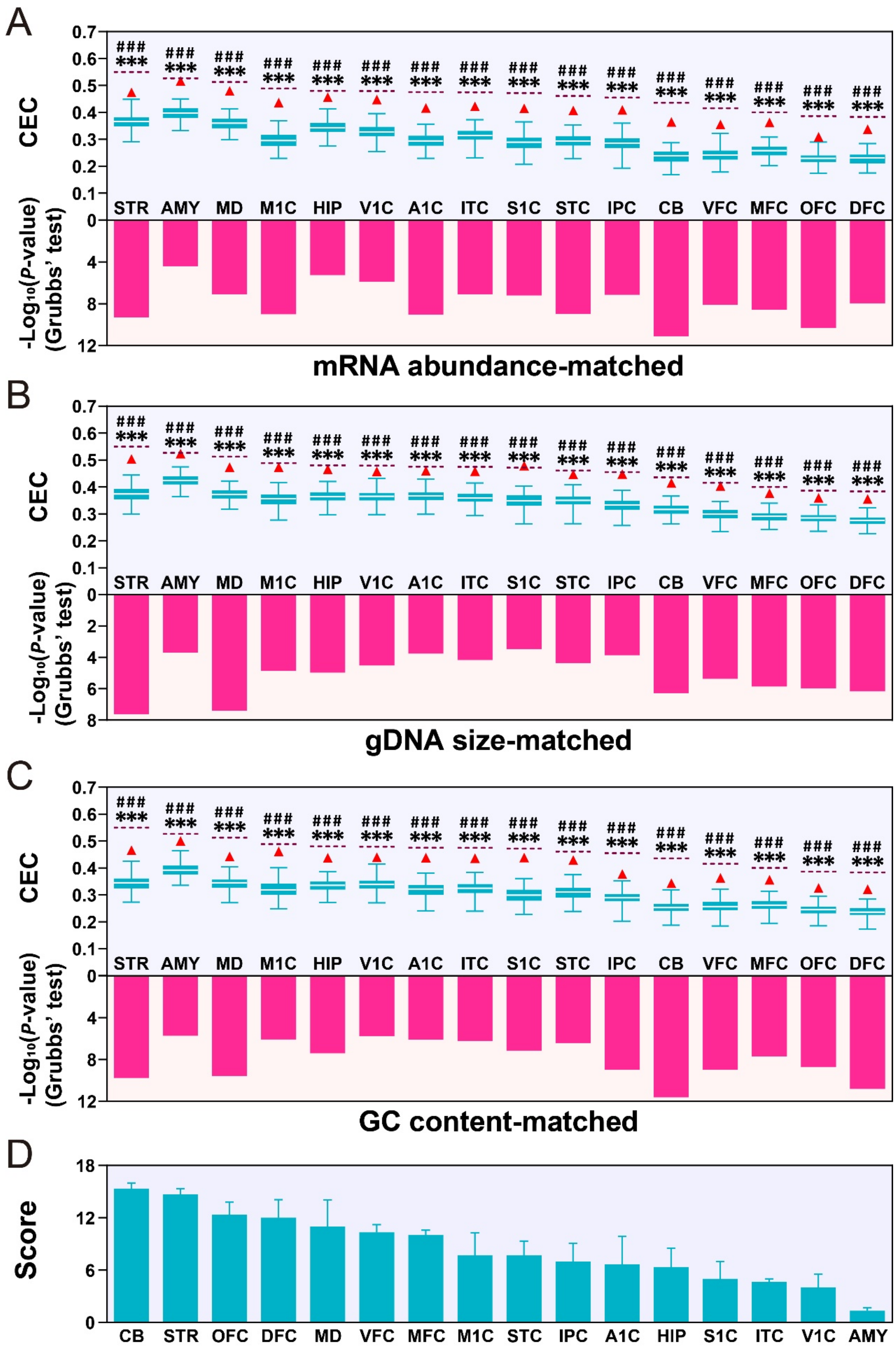
A
CEC
-Log10(P-value) (Grubbs' test)
STR AMY MD M1C HIP V1C A1C ITC S1C STC IPC CB VFC MFC OFC DFC
###
***
mRNA abundance-matched
B
CEC
-Log10(P-value) (Grubbs' test)
STR AMY MD M1C HIP V1C A1C ITC S1C STC IPC CB VFC MFC OFC DFC
gDNA size-matched
C
CEC
-Log10(P-value) (Grubbs' test)
STR AMY MD M1C HIP V1C A1C ITC S1C STC IPC CB VFC MFC OFC DFC
GC content-matched
D
Score
CB STR OFC DFC MD VFC MFC M1C STC IPC A1C HIP S1C ITC V1C AMY

Fig. S6 Co-expression of hcASDs in different brain regions. **A–C** Box plots showing the range of CECs of hcASD–mRNA and in different brain regions. **D** Significant scores of co-expression of hcASDs in different brain regions. Score = N − R where N is the number of regions and R is the integrated ranking (average of 3 conditions) of $-\log_{10}(P\text{-value})$ in Grubbs' test under four different ranking conditions.

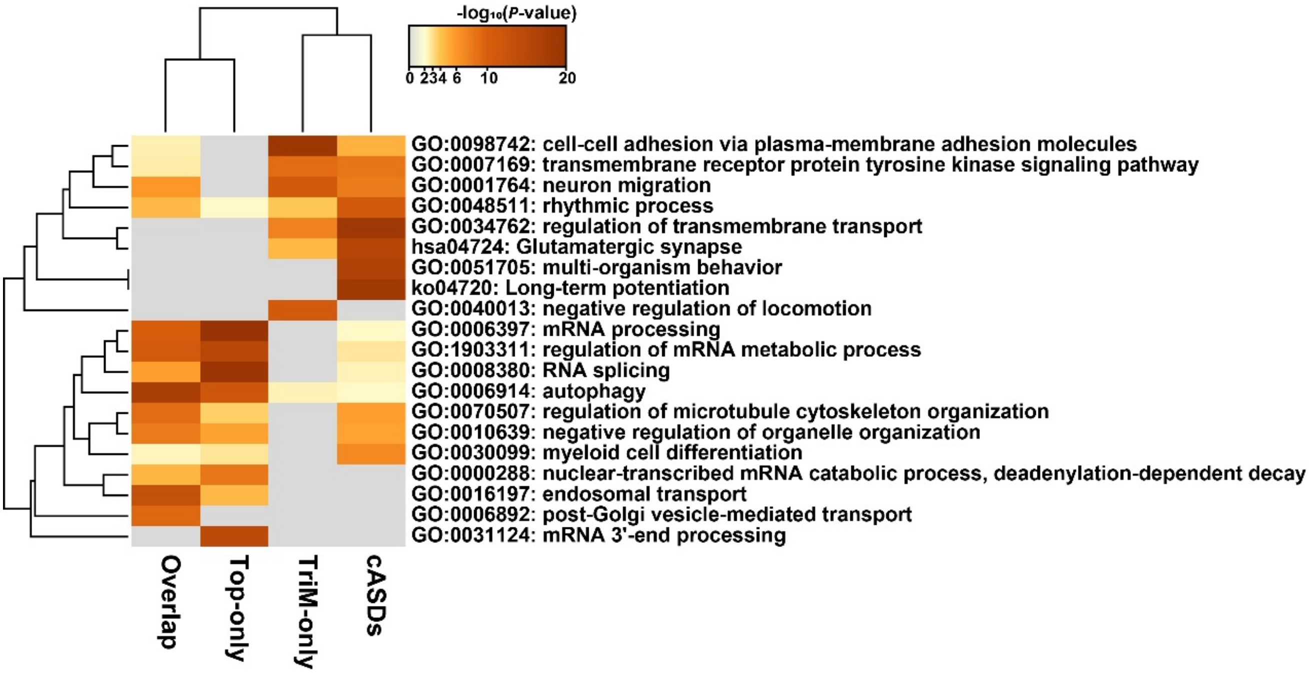

Fig. S7 Cluster analysis of $-\log_{10}(P\text{-value})$ in GO analysis of TriM-only, Top-only, Overlapped, and combined ASD gene sets (cASDs). $-\text{Log}_{10}(P\text{-value})$ values (0–20) are color-coded and shown in the heatmap. Boxes were colored according to their *P*-values. Gray boxes indicate a lack of enrichment for a specific GO term.
